# Endoscopic septotomy for symptomatic bariatric surgical complications: a new frontier of endoluminal surgery

**DOI:** 10.1055/a-2600-9952

**Published:** 2025-07-01

**Authors:** Abdulrahman Qatomah, Daryl Ramai, Christopher C. Thompson

**Affiliations:** 1 1861 Gastroenterology and Hepatology, Brigham and Womenʼs Hospital, Boston, United States; 2 195017 Gastroenterology and Hepatology, King Faisal Specialist Hospital and Research Centre – Jeddah, Jeddah, Saudi Arabia; 3 1861 Internal Medicine, Brigham and Womenʼs Hospital, Boston, United States; 4 1861 Division of Gastroenterology, Hepatology and Endoscopy, Brigham and Womenʼs Hospital, Boston, United States

Laparoscopic adjustable gastric banding (LAGB) and vertical band gastroplasty (VBG) are bariatric procedures that have declined in popularity due to long-term complications, including dysphagia secondary to esophageal dysmotility. Both procedures are associated with significant rates of reoperation or removal. Additionally, band erosion, though rare, is an established complication of LAGB that often manifests as chronic abdominal pain, dysphagia, and emesis 1 2 3 .

A 40-year-old man with a history of LAGB performed 20 years ago for weight loss which resulted in an eroded band, subsequently treated by surgical band removal and conversion to VBG due to weight regain. The patient developed gastric and esophageal dysmotility resulting in chronic abdominal pain and dysphagia that were not responsive to medical therapy. Upper endoscopy revealed post-VBG anatomy with a wide intragastric septum just distal to the LES causing an esophageal obstruction ( Fig. 1 ). Given the complexity of the surgical history, a multidisciplinary team determined that endoscopic therapy would be a feasible alternative to surgery.

**Fig. 1:**
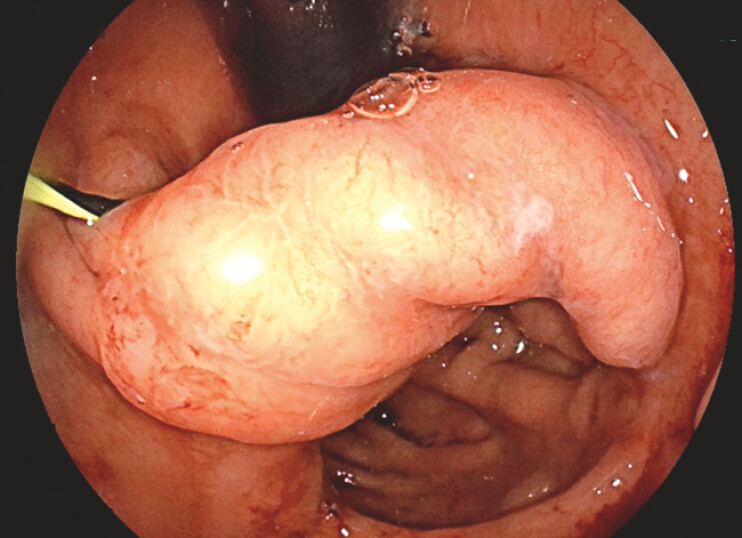
Intragastric septum on an endoscopic examination (with wire guidance) on retroflexion view.

Partial septotomy was initially attempted. Careful endoscopic ultrasound (EUS) ( Fig. 2 ) and tissue oxygenation assessment demonstrated normal oxygenation with no significant vascular structures within the septum. Submucosal incision and tunneling were performed to expose inter-septal tissue ( Fig. 3 ) followed by septotomy using a needle-type knife. Mucosal flaps were successfully closed with an endoscopic suturing device with a single running suture consisting of eight bites ( Video 1 ).

**Fig. 2:**
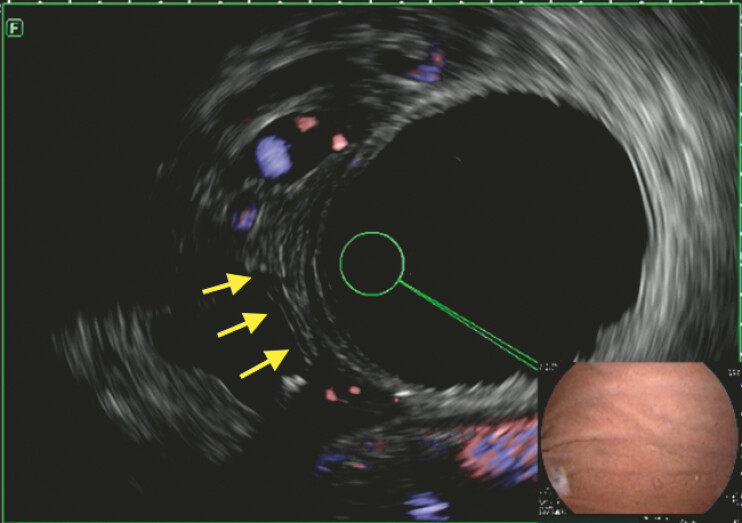
EUS examination with Doppler without major Doppler activity within septum. Abbreviation: EUS, endoscopic ultrasound.

**Fig. 3:**
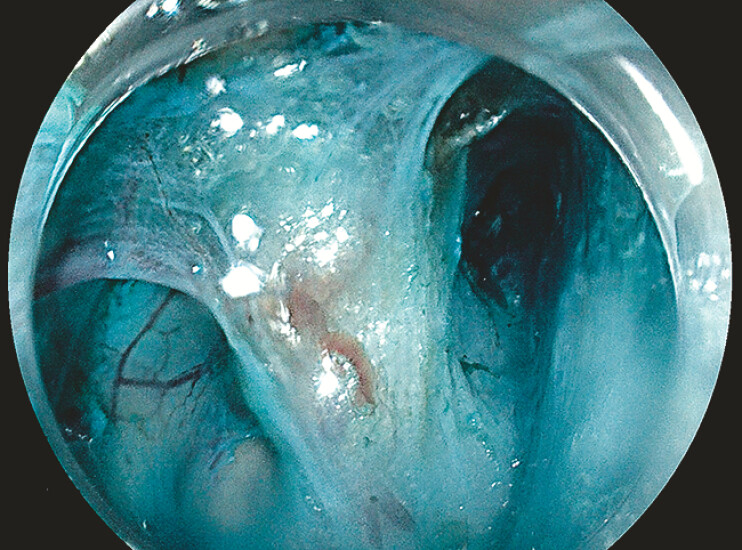
(video image): Inter-septal muscle exposed post submucosal tunneling.

Inter-septal muscle exposed post submucosal tunneling.Video 1

The patient experienced encouraging results, with some remaining symptoms, and wished to proceed with the completion septotomy. Repeat endoscopic and EUS examination demonstrated a reduced septal diameter without doppler-detectable vascular flow ( Fig. 4 ). Two large hemostatic clips were placed on the edges of the septum to prevent bleeding and perforation, followed by a successful complete septotomy using an insulated tip needle knife ( Fig. 5 ). To reinforce the edges and minimize bleeding risk, ligations were applied ( Video 1 ).

**Fig. 4:**
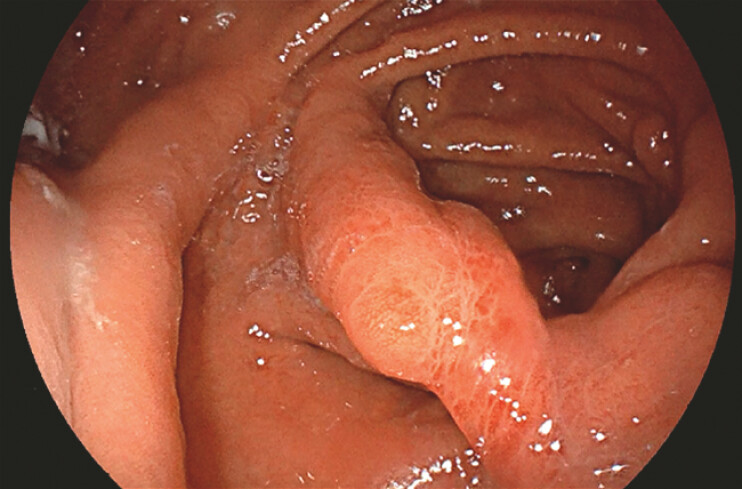
Follow-up endoscopy with a significant reduction in septum diameter.

**Fig. 5:**
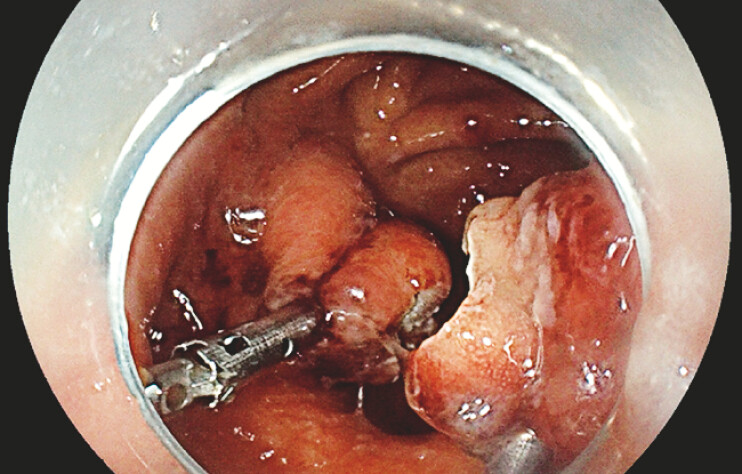
Appearance post complete septotomy.

Endoscopic septotomy represents a technically feasible and minimally invasive intervention for managing complications arising after bariatric surgery. The procedure demonstrates a favorable safety profile, contingent upon strict adherence to cautionary protocols, including vascular mapping and measures to mitigate the risk of perforation.

Endoscopy_UCTN_Code_TTT_1AO_2AN

## Notes

Endoscopy E-Videos https://eref.thieme.de/e-videos
*E-Videos* is an open access online section of the journal *Endoscopy* , reporting on interesting cases and new techniques in gastroenterological endoscopy. All papers include a high-quality video and are published with a Creative Commons CC-BY license. Endoscopy E-Videos qualify for HINARI discounts and waivers and eligibility is automatically checked during the submission process. We grant 100% waivers to articles whose corresponding authors are based in Group A countries and 50% waivers to those who are based in Group B countries as classified by Research4Life (see: https://www.research4life.org/access/eligibility/ ). This section has its own submission website at https://mc.manuscriptcentral.com/e-videos .
